# Effectiveness of nirsevimab against hospitalisation for RSV-bronchiolitis during high RSV-B circulation in the second year of nationwide implementation in France: a test-negative case-control study

**DOI:** 10.1016/j.lanepe.2025.101443

**Published:** 2025-09-10

**Authors:** Apolline Furgier, Camille Brehin, Corinne Levy, Romain Basmaci, Elise Launay, Camille Jung, Zein Assad, Léa Lenglart, Jérome Naudin, Anne-Lise Mary, Camille Aupiais, Loïc de Pontual, Valérie Biran, Béatrice Boutillier, Isabelle Hau, Mickael Shum, Sandra Biscardi, Céline Delestrain, Julie Toubiana, Jérémie F. Cohen, Amélie Lambert-Hoffert, Mathie Lorrot, Anne-Sophie Romain, Marion Ashman, Jee-Seon Yang, Blandine Prevost, Harriet Corvol, Clément Basse, François Dubos, Marie Cotillon, Constance Bridonneau, Lise-Martin Perceval, Etienne Bizot, Inès Fafi, Aurélie Portefaix, Léa Domitien, Carine Jaillet, Antoine Ouziel, Luigi Titomanlio, Stephane Bechet, Vincent Gajdos, Christèle Gras-Le Guen, Yves Gillet, Robert Cohen, Eric Jeziorski, Naim Ouldali, French Paediatric OVNI Network, Lola Alemende, François Angoulvant, Marta Bendavides, Aurélie Bourmaud, Stéphane Bonacorsi, Natacha Casanovas, Stéphane Dauger, Camille Delande, Claire Delcourt, Kadiatou Diallo, Anne Drouard, Xavier Durrmeyer, Benjamin Hallak, Mohamed M. El Hebil, Charlène Ferrandiz, Anna Giolito, Maud Gits, Géraldine Labouret, Olympe Marechal, Elodie Miha Nantenaina, Cecile Schrimpf, Perrine See, Zaba Valtuille, Sebastien Walser

**Affiliations:** a University of Paris Cite, Paris, France; b AP–HP, Department of General Pediatrics, Pediatric Infectious Disease, and Internal Medicine, Robert Debre Hospital, Paris, France; c Infection, Antimicrobials, Modelling, Evolution (IAME), INSERM UMR 1137, Paris Cité University, 75018, Paris, France; d French Paediatric Infectious Disease Group (GPIP), Nice, 06200, France; e Paediatric Infectious Diseases Department, Children's Hospital, Toulouse University Hospital, Toulouse, France; f Association Clinique et Therapeutique Infantile du Val-de-Marne France (ACTIV), France; g Association Française de Pédiatrie Ambulatoire (AFPA), Paris, France; h Université Paris Est, IMRB-GRC GEMINI, Créteil, France; i Paediatric Emergency Department and General Pediatrics Department, Louis Mourier University Hospital, Assistance Publique-Hôpitaux de Paris, Paris Cité Université, Colombes, France; j Paediatric Emergency Department and General Pediatrics Department Nantes Université, CHU Nantes, Pôle Hospitalo-Universitaire 5, Nantes, 44000, France; k Délégation de Recherche en Santé et Innovation University Paris Est Créteil, Créteil, France; l Paediatric Emergency Department, Robert Debré University Hospital, Assistance Publique-Hôpitaux de Paris, Paris Cité University, Paris, France; m Paediatric Intensive Care Unit, Robert-Debré University Hospital, AP-HP, Paris, France; n Sorbonne Paris Nord University, Assistance Publique-Hôpitaux de Paris, Paediatric Emergency Department and Department of General Paediatrics, Jean Verdier University Hospital, Bondy, France; o Neonatal Intensive Care Unit, Assistance Publique-Hôpitaux de Paris, Robert Debre Hospital, Paris, France; p General Pediatrics Department, Centre Hospitalier Intercommunal de Créteil, Créteil, France; q Necker Hospital for Sick Children, General Pediatrics and Pediatric Infectious Diseases, University of Paris Cité, Paris, France; r Department of General Pediatrics, Hôpital Armand-Trousseau, APHP, Université Paris Sorbonne, Paris, France; s Sorbonne University, Inserm U938, Saint-Antoine Research Center (CRSA), Assistance Publique – Hôpitaux de Paris (AP-HP), Trousseau Hospital, Paediatric Pulmonology Department, GRC SoLID, Paris, 75012, France; t Paediatric Emergency Unit and Infectious Diseases, Centre Hospitalier Universitaire Lille, Université de Lille, Lille, France; u Paediatric Emergency Department and Department of General Paediatrics, Antoine-Béclère Hospital-Assistance Publique-Hôpitaux de Paris, Paris Saclay University, Clamart, France; v Center of Clinical Investigations, INSERM CIC 1426, Robert-Debré University Hospital, Assistance Publique-Hôpitaux de Paris, Paris, 75019, France; w Hospices Civils de Lyon, Hôpital Femme Mère Enfant, Paediatric Intensive Care and Emergency Department, Bron, France; x Department of General Pediatrics, Infectiology, and Clinical Immunology, Department of Emergency, Post-Emergency Department, University Hospital of Montpellier, Montpellier, France; y KIDLIT Kids' Lyon Infectious Disease Team, Lyon, France

**Keywords:** Effectiveness, Nirsevimab, RSV-bronchiolitis, Test-negative study, Paediatrics

## Abstract

**Background:**

Nirsevimab was first introduced in September 2023, showing strong real-world effectiveness in preventing hospitalised RSV-bronchiolitis. RSV-A circulation predominated during 2023–2024, whereas RSV-B was frequent in 2024–2025. Recent reports indicated resistance to nirsevimab in RSV-B strains in France. We aimed to assess the effectiveness of large-scale nirsevimab implementation against hospitalised RSV-bronchiolitis during high RSV-B circulation.

**Methods:**

A test-negative case-control study was conducted using a national hospital-based surveillance system. All children <12 months hospitalised for bronchiolitis and tested for RSV in 12 French hospitals from October 10, 2024 to March 15, 2025, were included. Cases were RSV-positive bronchiolitis; controls were RSV-negative bronchiolitis. Effectiveness was assessed by a multivariable logistic regression model adjusted for confounders (sex, gestational age at birth, birth weight, risk factors for severe bronchiolitis, month of diagnosis, and medical centre). A range of sensitivity analyses was conducted.

**Findings:**

1270 patients were included; among which 830 (65.3%) were RSV-positive and 440 (34.7%) were RSV-negative bronchiolitis. The male-to-female sex ratio was 1.35, and the median age was 3 months (IQR: 1.5–5.6). Among cases, 182 (22.0%) received nirsevimab, compared to 282 (64.1%) for controls. Adjusted effectiveness against RSV-bronchiolitis was 84.9% (95% CI, 80.0–88.6). Subgroup analyses by age and severity (intensive care unit admission, respiratory support) showed consistent results, along with sensitivity analyses.

**Interpretation:**

Despite high RSV-B circulation, which has recently been identified to carry mutations potentially inducing resistance to nirsevimab, the effectiveness of the second national nirsevimab campaign against hospitalisation for RSV-bronchiolitis remained high.

**Funding:**

The study received financial support from 10.13039/100031283Sanofi, 10.13039/501100014088AstraZeneca, and the 2023 ATIP-Avenir grant from the 10.13039/501100001677National Institute of Health and Medical Research (Inserm).

**Research in context**

**Evidence before this study**

We conducted a PubMed search from inception up to February 2025 without language restriction using the following search terms: RSV, nirsevimab, and effectiveness.

Since the launch of nirsevimab immunisation campaigns in 2023–2024 to reduce RSV related morbidity, more than twenty studies have shown its early real-world effectiveness, estimated at around 70–85% in preventing both for hospitalised and non-hospitalised RSV-bronchiolitis. We did not identify any studies assessing the effectiveness of nirsevimab during the second immunisation campaign (2024–2025), particularly in France. A shift in RSV strain circulation occurred during the 2024–2025 season in France, with RSV-B becoming predominant, in contrast to RSV-A in 2023–2024. As RSV-B has recently been identified to carry rare mutations that could potentially reduce its sensitivity to nirsevimab, concerns have been raised regarding nirsevimab effectiveness for this second year of immunisation campaign.

**Added value of this study**

We conducted a test-negative study to evaluate the effectiveness of nirsevimab against hospitalised RSV-bronchiolitis in infants under 12 months in France during the second year of national immunisation programme (2024–2025). We found a strong nirsevimab effectiveness despite high RSV-B circulation.

**Implications of all the available evidence**

These findings add evidence regarding the sustained effectiveness of nirsevimab in the medium term, in the context of RSV-B circulation and widespread use of nirsevimab during a second national campaign, that may guide policymakers when recommending future immunisation campaigns.

## Introduction

Lower respiratory tract infections (LRTIs) are the leading cause of death in children under 5 years of age worldwide, accounting for 784,600 deaths in 2021.^1^ Respiratory syncytial virus (RSV) is considered the primary cause of LRTI in children, with an estimated 3.2 million hospital admissions worldwide each year among children under 5 years old.^2^

To prevent RSV infection, nirsevimab, a long-lasting recombinant monoclonal antibody binding the pre-fusion proteins of RSV has been recently developed.^3^ In France, nirsevimab was introduced in the 2023–2024 season,^4^ with an 80–83% effectiveness in preventing RSV-bronchiolitis in hospital inpatient settings, in paediatric emergency departments, and in ambulatory settings for that first season.^5^, ^6^, ^7^, ^8^, ^9^

Recent reports have identified few RSV variants with mutations in the binding site of the F protein, some of which potentially leading to resistance to nirsevimab. These mutations have all been observed in RSV-B strains.^10^

During the 2023–2024 RSV season, RSV-A was predominant in France, but for the 2024–2025 season, an important circulation of RSV-B has been observed,^11^ raising concerns about the risk of reduced nirsevimab effectiveness due to the potential circulation of nirsevimab-resistant strains. In this context, we aimed to estimate the effectiveness of nirsevimab against RSV-bronchiolitis requiring hospitalisation during the second national nirsevimab campaign in 2024–25.

## Methods

### Study design

We conducted a national prospective multicentric test-negative study between October 10, 2024 and March 15, 2025 in France. All children younger than 12 months hospitalised for bronchiolitis with an RSV nasopharyngeal test in one of the 12 participating centres during the study period were included.

Exclusion criteria were children previously immunised with palivizumab, those with a maternal history of RSV vaccination during pregnancy, those with a documented history of two or more wheezing episodes (considered as asthma), those who did not undergo an RSV test, incomplete data on nirsevimab immunisation or hospital stays of less than 24 h.

We defined cases as patients with bronchiolitis and a positive test for RSV, and controls as patients with bronchiolitis and a negative test for RSV. On the basis of national and international guidelines, bronchiolitis was defined as the first wheezing attack with respiratory symptoms before 12 months of age or the second attack in infants without a personal or family history of asthma or atopy.^12^ The RSV diagnostic test consisted either of a PCR test (BioFire Respiratory Panel and FilmArray BioMérieux®, SARS-CoV-2/FluA/B/RSV Assay®)^13^ with a sensitivity of 80–100% and a specificity of 100%,^14^^,^^15^ or of a rapid antigen test with a sensitivity of 79–90% and a specificity of 97–100%,^16^, ^17^, ^18^ both performed on upper airway samples. Patients with a positive test for both RSV and another respiratory virus were classified as cases. We considered a delay of less than 7 days between nirsevimab administration and hospital admission as equivalent to the absence of immunisation.

### Nirsevimab national campaign

The French authorities have issued recommendations to continue the national immunisation program with nirsevimab for a second year, 2024–2025 season^19^ for all children born after January 1, 2024, starting from September 15, 2024 and finishing on January 31, 2025. For the 2024–2025 season, immunisation coverage in France has been estimated at 84% in maternity wards and at 60% in outpatient settings.^20^ To increase protection against RSV, in addition to nirsevimab immunisation, a maternal vaccination campaign was launched in France on September 15, 2024, with Abrysvo®, achieving an estimated coverage of 30% among eligible pregnant women.^20^ Therefore, for the 2024–2025 season, the French guidelines recommended either maternal vaccination between 32 and 36 weeks of pregnancy or administration of nirsevimab during the first year of life.^19^

### Settings and data collection

The 12 participating centres were secondary and tertiary hospitals located across metropolitan France. Data collected included demographic, clinical, biological, and short-term outcome data.^13^

### Outcome and exposure measures

The primary outcome was hospitalisation for RSV-positive bronchiolitis. The main exposure factor of interest was RSV immunisation status with nirsevimab among case and control patients. The secondary outcomes included the need for ventilatory support and intensive care unit (ICU) admission. We conducted five subgroup analyses based on age (<3 months and ≥3 months), the presence of at least one risk factor for severe bronchiolitis (including congenital heart disease, bronchopulmonary dysplasia, or a gestational age <35 weeks for infants under 6 months), admission to the ICU, type of ventilatory support (oxygen therapy, non-invasive ventilation, or invasive ventilation) and type of test done (PCR or rapid antigenic test).

### Sample size calculation

On the basis of the hypothesis of a coverage for the immunisation by nirsevimab of 50% in France for this second year, the study sample size was calculated to detect a 50% reduction in the odds of nirsevimab immunisation among case patients. Assuming a two-sided α of 0.05 and a power of 0.90, 170 patients were needed in each group (i.e., at least 340 patients in total).

### Statistical analysis

The effectiveness of nirsevimab against RSV bronchiolitis was estimated using mixed-effects multivariable logistic regression (including a random effect for centres), adjusted for potential confounders, by comparing nirsevimab immunisation status between case and control patients. Effectiveness was calculated using the following equation: Effectiveness = 100% × (1 − adjusted odds ratio).

In light of current medical knowledge, the potential confounding variables selected included age at diagnosis, prematurity, birth weight, risk factors for severe bronchiolitis (including congenital heart disease, bronchopulmonary dysplasia, or a gestational age <35 weeks for infants under 6 months^6^), the month of bronchiolitis diagnosis, and the participating centre.

We performed multiple imputation by chained equations to handle missing data, generating 20 replicates. As recommended, the variables included in the imputation model were RSV infection status (binary), nirsevimab immunisation (binary), and all covariates used in the multivariable regression analysis.^21^ The results were combined using Rubin's rules.^22^

Various sensitivity analyses were conducted to assess the robustness of the main results. First, we performed an analysis without a random effect for centres using a multivariable logistic regression. Second, we developed a multivariable model excluding patients with one previous episode of bronchiolitis to minimise potential misclassification between bronchiolitis, recurrent wheezing, or other respiratory diseases. Third, we conducted a complete case analysis to explore the impact of missing data and multiple imputations. Fourth, we performed an additional analysis by converting continuous variables (term, birth weight, and age) into categorical variables. Fifth, we developed a multivariable model adjusted for the week of viral circulation to better account for rapid changes in RSV circulation. Sixth, we conducted a multivariable analysis without considering the 7-day delay between nirsevimab immunisation and hospitalisation. Seventh, to further account for indication bias, we performed a propensity score analysis using the overlap weighting method, employing a binomial model due to the overdispersion of values.^23^ Eighth, we conducted a multivariable analysis including only patients who received nirsevimab during the second year of the immunisation campaign, excluding those who had been vaccinated in the previous season (Supplementary Appendix Table S1).

The analyses were performed by R Studio v4.3.2. The main packages used were “dplyr”, “WeightIt”, “broom.mixed”, “mice”, “epitools”, “ggplot2”.

### Ethical approval

The study was approved by an ethics committee (CHI Creteil Hospital, France) and was registered at ClinicalTrials.gov (NCT06112132).

### Role of the funding source

The funders of the study had no role in the study design, data collection, data analysis, or data interpretation.

## Results

### Characteristics of the case and control patients

**Fig. 1:**
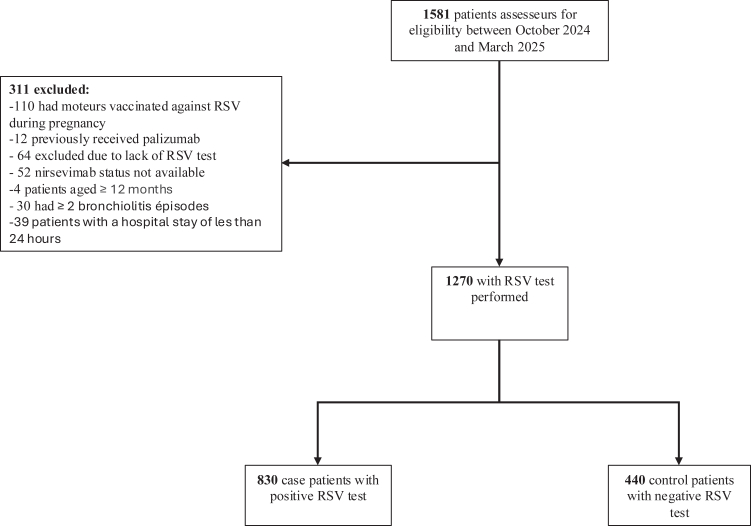
**Flowchart of study participants.** Abbreviation: RSV = respiratory syncytial virus.

Among 1581 eligible patients, 311 were excluded (Fig. 1), and 1270 children were included in the analysis. 29 infants who received nirsevimab less than seven days before admission were recoded as not exposed.

RSV PCR was performed for 1149 patients, and RSV antigen testing was conducted for 98 patients. Among the included patients, 830 (65.4%) tested positive for RSV (cases), and 440 (34.6%) tested negative (controls). The median age of the study population was 3 months (IQR: 1.5–5.6). By group, the median age and sex ratio were similar (Table 1). There were more preterm infants in the control group, but the median age at birth was similar, with respectively 39 GA (IQR: 38–40) and 38 GA (IQR: 37–40). The two groups were comparable regarding risk factors for severe bronchiolitis. Co-detection of other viruses with RSV was observed in 176 patients (21.2%) in the case group. In the control group, the main virus identified was rhinovirus/enterovirus (141 (32.2%)). Admission centre and month were also comparable, with a peak in October and December (Figure S2 and S3, Supplementary Appendix).

**Table 1:** Characteristics of cases (RSV positive) and control (RSV negative) groups.

Variables	RSV positive bronchiolitis (cases) (n = 830)	RSV negative bronchiolitis (controls) (n = 440)
**Demographics data**		
**Age at admission:** median (IQR)–month	3 (1–5)	2 (1–5)
0–2 month	402 (48.4)	223 (50.7)
3–5 month	306 (36.9)	141 (32.0)
>6 months	122 (14.7)	76 (17.3)
**Sex assigned at birth ratio:** M/F	456/374	259/181
**Gestational age at birth:** median (IQR)–week	39 (38–40)	38 (37–40)
**Term birth:** no. (%)		
<32 GA	16 (1.9)	32 (7.3)
32–36 GA	61 (7.3)	54 (12.3)
≥37 GA	691 (83.2)	321 (72.9)
*NA*	62 (7.6)	33 (7.5)
**Birth weight:** no. (%)–grams		
<1000	8 (1.0)	14 (3.2)
1000–2500	52 (6.2)	67 (15.2)
>2500–<4000	643 (77.5)	305 (69.3)
≥4000	38 (4.6)	16 (3.7)
*NA*	89 (10.7)	38 (8.6)
**History**		
1 previous history of bronchiolitis: no./total no. (%) (NA = 12)	56 (6.7)	51 (11.6)
**Risk factor for severe bronchiolitis:** no./total no. (%)		
Preterm birth (<35 GA) and age of <6 month	29 (3.5)	31 (7.0)
Bronchopulmonary dysplasia	7 (0.8)	16 (3.6)
Congenital heart disease	10 (1.2)	5 (1.1)
**Medical care**		
Duration of hospitalisation: median (IQR)–days	4.0 (2.0–6.0)	3.0 (2.0–5.0)
ICU admission: no./total no. (%)	139 (16.7)	46 (10.4)
Supplemental oxygen use: no./total no. (%)	485 (59.5)	171 (42.2)
Non-invasive ventilation: no./total no. (%) (NA = 1)	113 (13.9)	33 (8.2)
Invasive ventilation: no./total no. (%) (NA = 1)	3 (0.4)	0
**Type of diagnostic test performed:** no./total no. (%)		
PCR test	763 (91.9)	409 (93.0)
Rapid antigenic test	67 (8.1)	31 (7.0)
**Other respiratory virus detected:** no./total no. (%) (NA = 53)	176 (21.2)	281 (63.9)
*Influenza*	8 (1.0)	54 (12.3)
*Rhinovirus/enterovirus*	129 (15.5)	141 (32.0)
*Parainfluenza*	7 (0.8)	34 (7.7)
*SARS-CoV-2*	8 (1.0)	16 (3.6)
*Adenovirus*	32 (3.8)	29 (6.6)
*Metapneumovirus*	8 (1.0)	70 (15.9)
**Month of diagnosis:** no./total no. (%)		
October 2024	62 (7.5)	53 (12.0)
November 2024	340 (41.0)	126 (28.6)
December 2024	333 (40.1)	127 (28.9)
January 2025	76 (9.2)	85 (19.3)
February 2025	17 (2.0)	49 (9.5)
March 2025	2 (0.2)	7 (1.6)

Abbreviations: RSV = respiratory syncytial virus, no = number (of patients), NA = not available, missing data, M/F = ratio male to female, GA = gestational age, IQR = inter quartile range = First Quartile and Third Quartile, ICU = Intensive Care Unit.

### Primary outcome

**Fig. 2:**
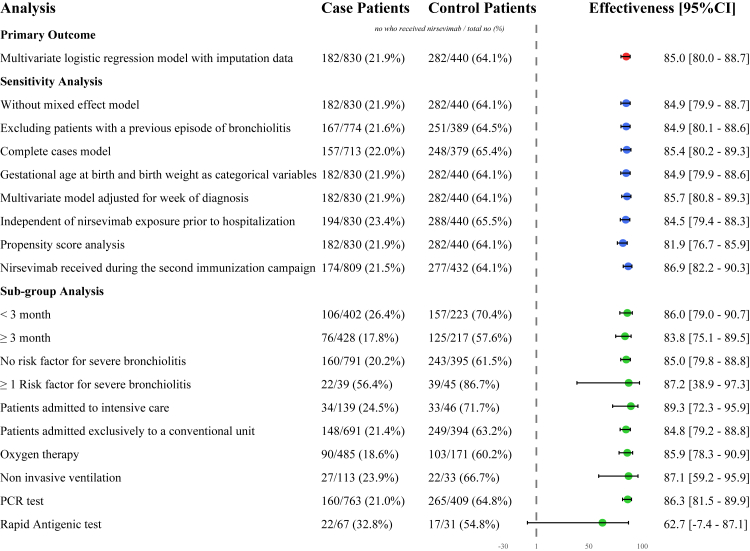
**Effectiveness of nirsevimab for hospitalised bronchiolitis.** Case and control patients are infants with bronchiolitis hospitalised with respectively an upper way test positive for RSV or negative. n/N (%) represents the number of infants immunised with nirsevimab divided by the total number of case or control patients. Effectiveness were calculated using the following formula: (1 − adjusted odds ratio) × 100. Multiple imputation was used for all sensitivity analysis, except for the complete cases model. All the details of the differences analysis are detailed in Table S1 in Supplementary Appendix. Multivariate logistic regression model was adjusted for age (in month), prematurity (yes/no), birth weight (in kilogrammes), risk factor of severe bronchiolitis (including congenital heart disease, bronchopulmonary dysplasia, or a gestational age <35 weeks for infants under 6 months (yes/no)), month of bronchiolitis diagnosis, and the participating centre. Abbreviation: CI = confidence interval.

In the study population, 464 were immunised by nirsevimab: 182 (39.2%) in the case group (RSV positive) and 282 (60.8%) in the control group (RSV negative). The adjusted effectiveness of nirsevimab against hospitalisation for RSV-bronchiolitis was 84.9% (95% confidence interval [CI], 80.0–88.6). Results from sensitivity analyses were similar (Fig. 2).

### Subgroup analysis

The effectiveness of nirsevimab for infants aged <3 months and ≥3 months was 86.0% (CI, 79.0–90.7) and 83.8% (CI, 75.1–89.5), respectively (Fig. 2). The effectiveness was also similar in those with one or more risk factors for severe bronchiolitis compared to those without, with 85.0% (CI, 79.8–88.8) and 87.2% (CI, 38.9–97.3). The effectiveness was also similar across oxygen therapy, non-invasive ventilation, and the type of test performed (PCR or rapid antigen test).

## Discussion

In this real-life study, we observed 84.9% (95% CI, 80.0–88.6) effectiveness of RSV immunisation with nirsevimab in preventing RSV bronchiolitis-related hospitalisation in young infants during the second national campaign (2024–2025). These results were confirmed by several sensitivity analyses. During the first year of nirsevimab implementation (2023–2024), a significant effectiveness had been demonstrated at all stages of the disease (from ambulatory care to intensive care admission) worldwide.^5^, ^6^, ^7^, ^8^, ^9^^,^^13^^,^^24^, ^25^, ^26^, ^27^, ^28^ Thus, our results from the second campaign (2024–2025) showed that effectiveness of nirsevimab was not eroded by a shift in virus strains circulation compared to the first season (2023–2024).

A change occurred in the strain circulation of RSV, particularly in France. During the first season (2023–2024), viral circulation surveillance showed a major predominance of RSV-A, with a peak at around 88% in December 2023.^11^ However, in the second year of national immunisation (2024–2025), RSV-B predominated, peaking at around 90% in September 2024 and accounted for approximately 60–70% overall during the study period,^11^ raising concerns about a potential surge of nirsevimab-resistant strains. Indeed, Fourati et al. found in the largest genotypic and phenotypic French surveillance study few RSV-B strains which carried mutations (F:N208D and F:I64M/K65E) that were potentially responsible for a more than 500-fold reduction in fusion inhibition.^10^ Several mutations involving the nirsevimab binding sites that could potentially alter nirsevimab function have been described previously.^29^^,^^30^

The clinical consequences of these potential resistance to nirsevimab remain unknown. Furthermore, the capacity of these resistant strains to emerge and circulate in the population remains unknown to date, and depends in particular on the fitness of these clones and the selection pressure brought about by the widespread use of nirsevimab. While the fitness of these strains remains unexplored to date, our data suggest that, despite the widespread use of nirsevimab (with an estimated coverage of approximately 80% in infants under 12 months^20^), these resistant strains have not emerged widely. This is reassuring regarding the strong protection nirsevimab provides against RSV-related hospitalised bronchiolitis in infants.

These results support the implementation of the national nirsevimab immunisation campaign in France. Although a few RSV-related hospitalisations were recorded after the end of the campaign, the number of included participants (cases and controls combined) was low in February and March, suggesting that extending the campaign beyond January 31 would have not provided an important additional benefit.

This study has several limitations which do not allow for causative conclusions.

First, due to the non-randomised design, a causative conclusion cannot be drawn. However, the test-negative design, which has been widely used to assess vaccine effectiveness, including recent studies for RSV, offers a validated approach to evaluate the real-world immunisation effectiveness,^5^^,^^24^ notably by mitigating bias related to healthcare-seeking behaviour.^31^

Second, we were not able to determine the strains (RSV-A and RSV-B) in our study. However, the strains sent to the National Reference Center were classified accordingly, showing a predominance of RSV-B during the study period.^11^ Further sequencing and/or viral subtyping is needed to better assess the proportion of RSV-A and RSV-B circulating in France and, more broadly, in Europe. Third, false positive or negative RSV test results can occur. To assess the potential impact of classification bias, we conducted a subgroup analysis by test type. One subgroup included patients who underwent a PCR test, while the other included those who had a rapid antigen test, with both groups yielding similar results in terms of nirsevimab effectiveness. The number of co-infections may be underestimated due to the lack of large-scale multiplex PCR testing in all centres; therefore, these data should be interpreted with caution. Fourth, our study may have been influenced by an indication bias which could lead to underestimation of the true effectiveness of the immunisation. Indeed, there may have been an overrepresentation of immunised children due to their underlying conditions, which placed them at higher risk for severe bronchiolitis. This hypothesis is supported by the comparison of the overall characteristics of immunised and non-immunised patients, which slightly differed for the bronchiolitis risk factors variable (Table S2, Supplementary Appendix). However, the subgroup analysis based on the presence or absence of a risk factor revealed similar effectiveness, although with a wide confidence interval due to the small sample size. To further investigate this potential indication bias, we applied a propensity score with overlap weighting, which produced similar results. Thus, despite a potential indication bias, the effectiveness of nirsevimab in our study remains high. Of note, propensity score analysis reports marginal, rather than conditional effects. Fifth, the role of inequalities including socioeconomic status and ethnicity was not explored in this study. Further studies are needed to analyse the role of this determinant.

### Conclusion

This real-world study evaluated the effectiveness of nirsevimab during the second year of immunisation in France (2024–2025). Despite the circulating RSV strain differing from that of the first year, the effectiveness remains high, providing robust protection for infants against RSV bronchiolitis. Further studies are needed to assess the long-term effectiveness of nirsevimab in the context of circulating resistant strains.

## Contributors

AF and NO take responsibility for the content of the manuscript, including the integrity of the data, the accuracy of the data analysis and were responsible for the decision to submit the manuscript. AF and NO have accessed and verified the data. NO conceptualised the study. AF led the literature review. AF and NO led the data analysis and visualisation. AF and NO led the data interpretation. AF and NO drafted the manuscript. RC, RB, CL, and EJ commented on the draft report. All authors contributed to critical revision of the manuscript for important intellectual content. All authors had full access to all the aggregated data in the study. All authors read and approved the final draft of the manuscript and had final responsibility for the decision to submit for publication. All authors of the French paediatric OVNI network contributed to critical revision of the manuscript for important intellectual content, read and approved the final draft of the manuscript.

## Data sharing statement

Anonymised data are available on reasonable request to the principal investigator (NO).

## Declaration of interests

RB declares receiving fees from Sanofi and MSD for medical conferences or scientific meetings. CL declares receiving travel grants from MSD, Pfizer and fees from Sanofi, GSK, MSD, and Pfizer for scientific meetings and expert board participation. RC reports personal fees and non-financial support from Pfizer and personal fees from Cerballiance, GSK, Merck, Pfizer, Sanofi, Viatris outside the submitted work. NO declares receiving travel grants from MSD, Pfizer, Sanofi, and GSK. VG declares receiving fees from Sanofi and MSD for medical conferences and expert group. EL declares receiving travel grants from GSK and fees for medical conference from MSD. EJ declares receiving travel grants from Sanofi, GRIFOLS, MSD and fees from Sanofi, LFB, GSK, Pfizer for scientific meetings and expert board participation. IH declares receiving grants drop Pfizer, Sanofi, and MSD, and consulting fees from Cerballiance and payment for symposiums from GSK. FD déclares receiving travel Grant from MSD and being board member for Sanofi and MSD. All other authors have no conflicts of interest to disclose.
