# Supplementary material for: Effectiveness of nirsevimab against hospitalisation for RSV-bronchiolitis during high RSV-B circulation in the second year of nationwide implementation in France: a test-negative case-control study
Source: Lancet Reg Health Eur. 2025 Sep 10;58:101443. doi: 10.1016/j.lanepe.2025.101443 (PMC12598395; doi:10.1016/j.lanepe.2025.101443)

Supplementary Appendix

[**Table S1:** Sensitivity analyses for the effectiveness of nirsevimab against respiratory syncytial virus (RSV)-bronchiolitis. 2](#_Toc200728568)

[**Table S2:** Characteristics of patients excluded 3](#_Toc200728569)

[**Table S3:** Characteristics of immunized versus non-immunized patients (nirsevimab) 4](#_Toc200728570)

[**Table S4:** Descriptive statistics on the delay between nirsevimab immunisation and sample positivity 5](#_Toc200728571)

[**Figure S1**: Covariate balance for the propensity score analysis and balance measures 6](#_Toc200728572)

[**Figure S2**: Number of cases and controls by center 7](#_Toc200728573)

[**Figure S3:** Weekly number of case and control patients included over the study period 8](#_Toc200728574)

## **Table S1:** Sensitivity analyses for the effectiveness of nirsevimab against respiratory syncytial virus (RSV)-bronchiolitis.

| **Identification number of the analysis** | **Sensitivity analysis** | **Characteristics of the model** | **Explanatory variables** | **Comments** |
| --- | --- | --- | --- | --- |
| 2 | Without mixed effect | Multivariable logistic  regression model with multiple imputation and remove the mixed effect model. | - Nirsevimab immunization status: dichotomous  - Age of the infant at diagnosis: continuous  - Birth weight: continuous  - Prematurity: dichotomous  - Sex: dichotomous  - Risk factors for severe bronchiolitis: dichotomous  - Inclusion centre: categorial  - Month of diagnosis: categorial | All patients included |
| 3 | Multivariable model excluding patients with history of bronchiolitis | Multivariable logistic regression model with multiple imputation. | - Nirsevimab immunization status: dichotomous  - Age of the infant at diagnosis: continuous  - Birth weight: continuous  - Prematurity: dichotomous  - Sex: dichotomous  - Risk factors for severe bronchiolitis: dichotomous  - Inclusion center: categorial  - Month of diagnosis: categorial | Patient with history of previous bronchiolitis excluded. |
| 4 | Complete case analysis | Multivariable logistic regression model | - Nirsevimab immunization status: dichotomous  - Age of the infant at diagnosis: continuous  - Birth weight: continuous  - Prematurity: dichotomous  - Sex: dichotomous  - Risk factors for severe bronchiolitis: dichotomous  - Inclusion center: categorial  - Month of diagnosis: categorial | Patients with missing data for all covariates included in the model were excluded. |
| 5 | Transformation of continuous variables into categorial variables | Multivariable logistic regression model with multiple imputation. | - Nirsevimab immunization status: dichotomous  - Age of the infant at diagnosis: categorial (0–2-month, 3-6 month, ≥ 6 month)  - Birth weight: categorial (< 1000g,1000 -2500g, 2500 - < 4000g, ≥ 4000g)  - Birth term: categorial (<32 SA, 32-36 SA  ≥ 37 SA)  - Sex: dichotomous  - Risk factors for severe bronchiolitis: dichotomous  - Inclusion center: categorial  - Month of diagnosis: categorial | Birth term and birth weight as categorical variables instead of continuous variables |
| 6 | Multivariable model adjusted for week of diagnosis | Multivariable logistic regression model with multiple imputation. | - Nirsevimab immunization status: dichotomous  - Age of the infant at diagnosis: continuous  - Birth weight: continuous  - Prematurity: dichotomous  - Sex: dichotomous  - Risk factors for severe bronchiolitis: dichotomous  - Inclusion center: categorial  - Week of diagnosis | Adjusted for the week of diagnosis to better account for the rapidly increasing national coverage of nirsevimab and the circulation of RSV over the study period. |
| 7 | Multivariable model disregarding the time between immunization and bronchiolitis hospitalization. | Multivariable logistic regression model with multiple imputation. | - Nirsevimab immunization status: dichotomous  - Age of the infant at diagnosis: continuous  - Birth weight: continuous  - Prematurity: dichotomous  - Sex: dichotomous  - Risk factors for severe bronchiolitis: dichotomous  - Inclusion center: categorial  - Month of diagnosis: categorial | The analysis was expanded to include cases of bronchiolitis that occurred within seven days of Nirsevimab administration. But patients who were administered nirsevimab during hospitalization were considered non-immunized. |
| 8 | Propensity score | Multivariable logistic regression model with multiple imputation and propensity score with the overlap weighting method | - Nirsevimab immunization status: dichotomous  - Age of the infant at diagnosis: continuous  - Birth weight: continuous  - Prematurity: dichotomous  - Sex: dichotomous  - Risk factors for severe bronchiolitis: dichotomous  - Inclusion center: categorial  - Month of diagnosis: categorial | Overlap weighting (OPW) method |
| 9 | Multivariable model for patients immunized during the second national campaign. | Multivariable logistic regression model with multiple imputation. | - Nirsevimab immunization status: dichotomous  - Age of the infant at diagnosis: continuous  - Birth weight: continuous  - Prematurity: dichotomous  - Sex: dichotomous  - Risk factors for severe bronchiolitis: dichotomous  - Inclusion center: categorial random  - Month of diagnosis: categorial | Patients immunized with nirsevimab before 15/07/2024 and patients who were born before 31/01/2024 ; and had missing immunization dates were excluded from the analysis. |

## **Table S2:** Characteristics of patients excluded

| VARIABLES | Excluded Patients  (N=311) | Included Patients  (N=1270) |
| --- | --- | --- |
| Sex assigned at birth ratio: M/F | 175/136 | 715/555 |
| Age at admission: median (IQR) -month | 2 (1-6) | 3 (1-2) |
| Gestational age at birth: median (IQR) - week | 39 (38-40) | 39 (38-40) |
| Birth weight: median (IQR) – grams | 3230 (3862-3547) | 3220 (2840-3575) |
| Immunisation par Nirsevimab : no. /total no. (%) |  |  |
| Yes | 65 (27.0) | 778 (61.2) |
| No | 175 (73.0) | 492 (38.8) |
| RSV-bronchiolitis: no./total no. (%) |  |  |
| Yes | 130 * (55.3) | 830 (65.3) |
| No | 105 (44.7) | 440 (34.6) |
| Médical care : |  |  |
| Duration of hospitalization: median (Q1-Q3) - days | 2 (1-5) | 3 (2-5) |
| ICU admission: no./total no. (%) | 32 (10.3) | 185 (14.6) |
| Supplemental oxygen use: no./total no. (%) | 153 (49.2) | 656 (51.6) |
| Ventilatory support: no./total no. (%) | 22 (7.07) | 146 (11.5) |
| Invasive ventilation: no./total no. (%) | 3 (1.0) | 1(0.1) |

Abbreviation:

M/F = ratio male to female

IQR = interquartile range = First Quartile and Third Quartile

no or n = number

RSV = respiratory syncytial virus

ICU = Intensive Care Unit

*NA= not available = 76

** NA = 71

## **Table S3:** Characteristics of immunized versus non-immunized patients (nirsevimab)

| VARIABLES | Patients immunized  (N=464) | Patients non immunized  (N=804) |
| --- | --- | --- |
| Sex assigned at birth ratio: M/F | 279/185 | 436/370 |
| Sex assigned at birth ratio: M/F | 2 (1-4) | 3 (1-5) |
| Age at admission: median (IQR) -month | 39 (37-40) | 39 (38-40) |
| Gestational age at birth: median (IQR) - week | 3130 (2662-3500) | 3294 (2927-3611) |
| Birth weight: median (IQR) – grams | 61 (13.1) | 23 (2.9) |
| Preterm birth (<35 GA) and age of < 6 month | 42 (9.1) | 18 (2.2) |
| Pulmonary dysplasia | 22 (4.7) | 1 (0.1) |
| Congenital heart disease | 10 (2.2) | 5 (0.6) |
| Médical care : |  |  |
| Duration of hospitalization: median (Q1-Q3) - days | 3 (2-5) | 4 (2-5) |
| ICU admission: no./total no. (%) | 67 (14.4) | 118 (14.7) |
| Supplemental oxygen use: no./total no. (%) | 193 (41.6) | 463 (57.6) |
| Ventilatory support: no./total no. (%) | 49 (10.6) | 97 (12.1) |
| Invasive ventilation: no./total no. (%) | 1 (0.2) | 2 (0.2) |

Abbreviation:

M/F = ratio male to female

IQR = interquartile range = First Quartile and Third Quartile

no or n = number

RSV = respiratory syncytial virus

ICU = Intensive Care Unit

## **Table S4:** Descriptive statistics on the delay between nirsevimab immunisation and sample positivity

*887 missing data

|  | **Minimum** | **Q1** | **Median** | **Mean** | **Q3** | **Maximum** |
| --- | --- | --- | --- | --- | --- | --- |
| **Delay between nirsevimab immunisation and sample positivity (in days)*** | 0 | 27 | 46 | 59 | 75 | 360 |

## **Figure S1**: Covariate balance for the propensity score analysis and balance measures


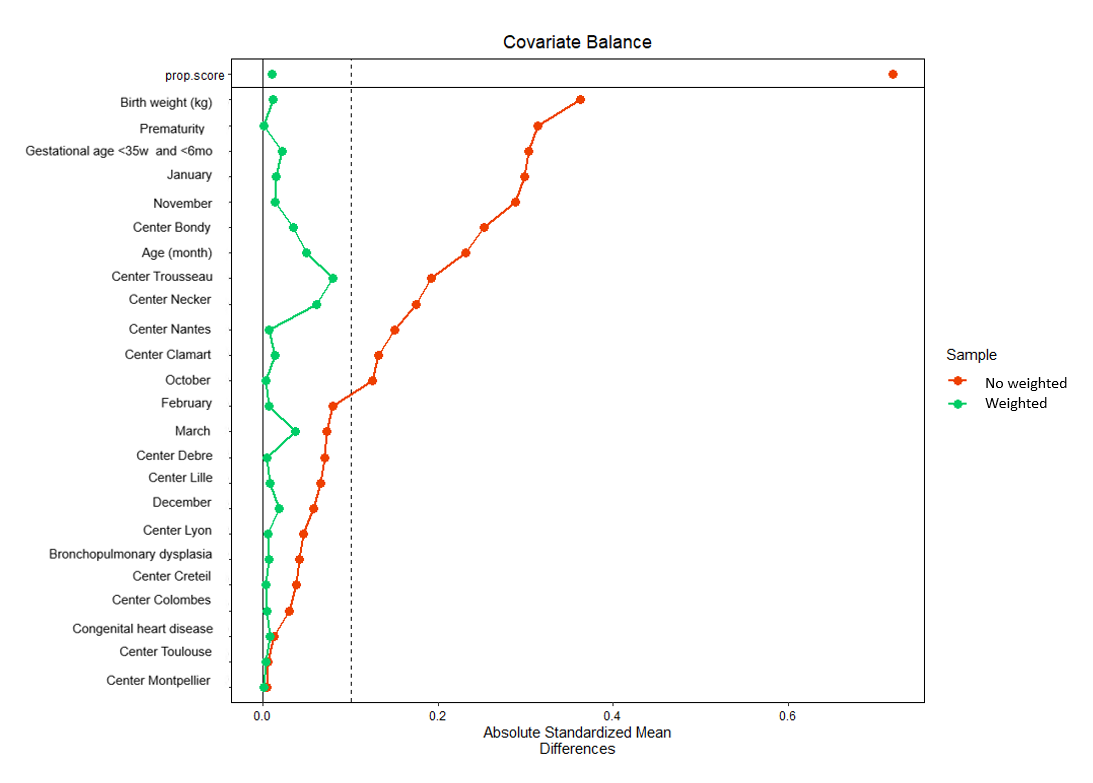


| **Balance measures** | **Type** | **Standardized difference after adjustment** |
| --- | --- | --- |
| Propensity score | distance | -0.0099 |
| age (month) | continue | 0.0794 |
| prematurity | binary | 0.0001 |
| weight (kg) | continue | -0.0111 |
| bronchopulmonary dysplasia | binary | 0.0033 |
| congenital heart disease | binary | -0.0016 |
| gestational age <35w and 6 month | binary | -0.0034 |
| centre Bondy | binary | -0.0092 |
| centre Creteil | binary | -0.0009 |
| centre Clamart | binary | 0.0016 |
| centre Colombes | binary | 0.0020 |
| Centre Debre | binary | -0.0073 |
| Centre Lille | binary | 0.0013 |
| Centre Lyon | binary | -0.0009 |
| Centre Montpellier | binary | -0.0002 |
| Centre Nantes | binary | 0.0007 |
| Centre Necker | binary | 0.0016 |
| Centre Toulouse | binary | 0.0009 |
| Centre Trousseau | binary | 0.0103 |
| January | binary | -0.0045 |
| February | binary | 0.0010 |
| March | binary | 0.0007 |
| October | binary | -0.0104 |
| November | binary | 0.0164 |
| December | binary | -0.0032 |

## **Figure S2**: Number of cases and controls by center


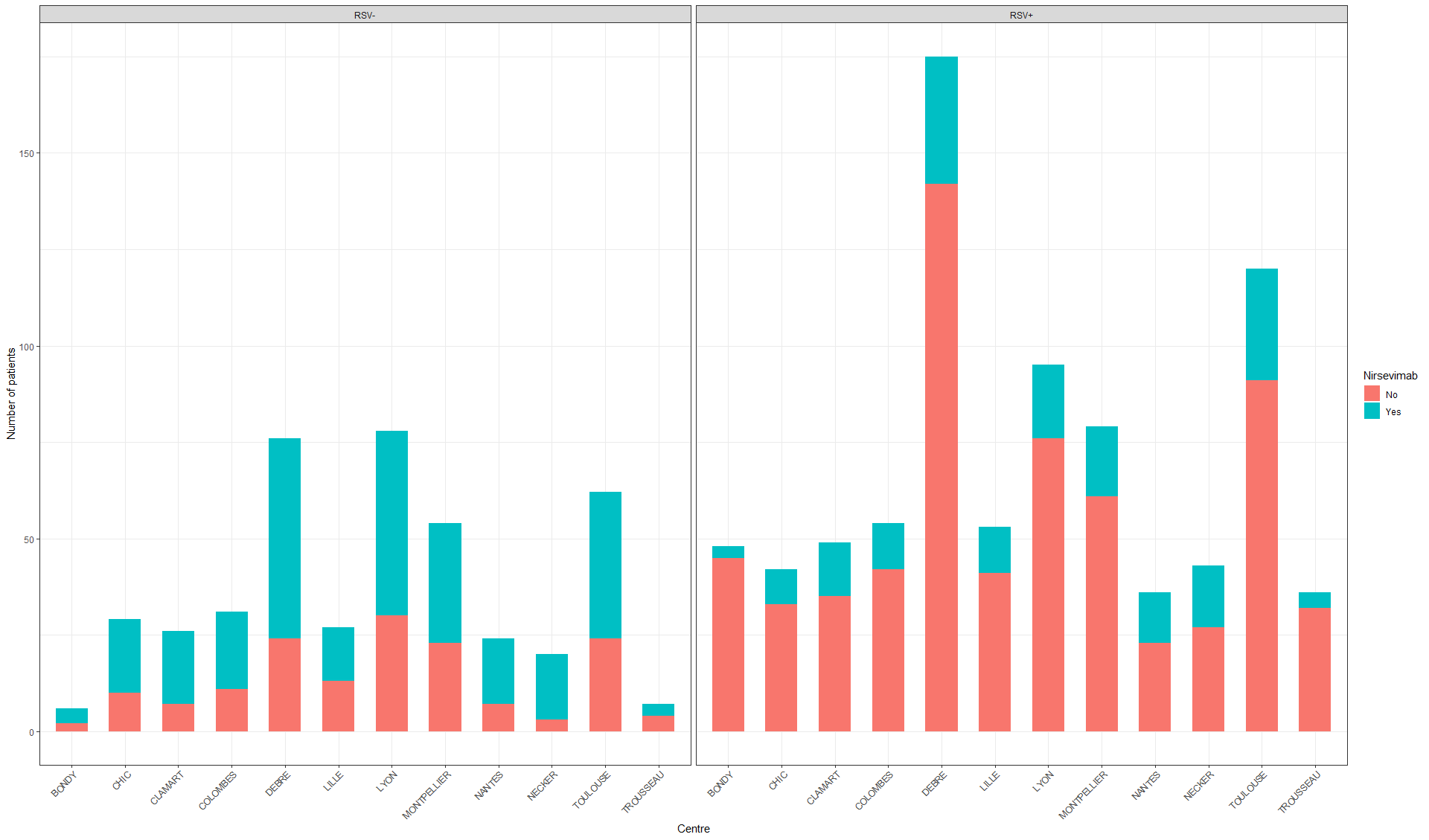


## **Figure S3:** Weekly number of case and control patients included over the study period


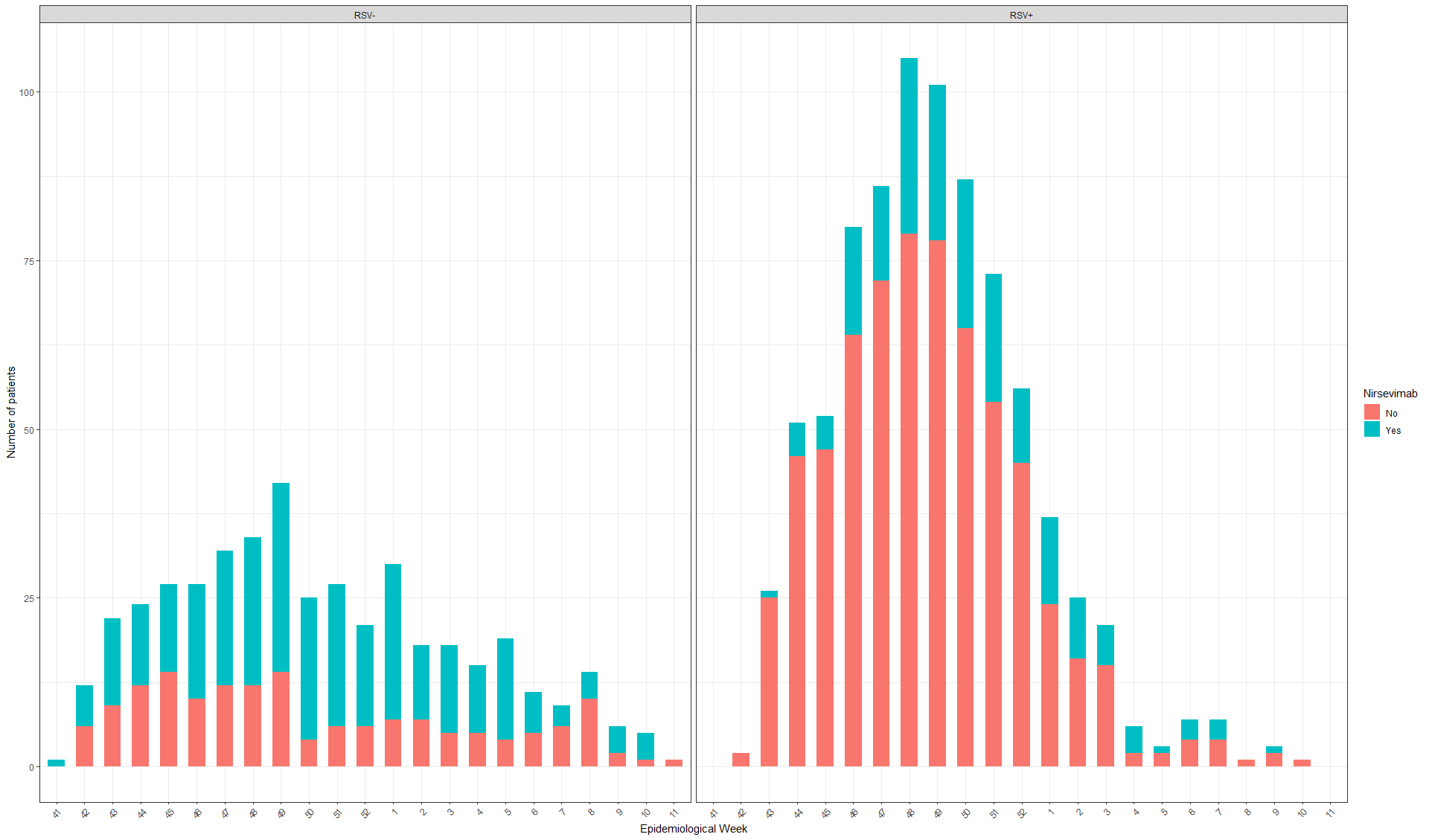

Supplement: Supplementary Appendix-Tables and Figures [file mmc1.docx]
